# A stepwise institutional approach to refractory autoimmune cytopenia after allogeneic stem cell transplantation in children: a retrospective case series

**DOI:** 10.3389/fimmu.2026.1902584

**Published:** 2026-09-17

**Authors:** Bei Yan, Yuan Sun, Zhouyang Liu, Jiao Chen, Shifen Fan, Fan Jiang, Linlin Lv

**Affiliations:** Department of Hematology and Oncology, Beijing JingDu Children’s Hospital, Beijing, China

**Keywords:** allogeneic hematopoietic stem cell transplantation, autoimmune cytopenia (AIC), immune reconstitution, immune tolerance, immunomodulation, regulatory T cells

## Abstract

**Background:**

Refractory autoimmune cytopenia (AIC) after allogeneic hematopoietic stem cell transplantation carries substantial morbidity and mortality in children. Conventional therapy fails in approximately half of patients, and escalating immunosuppression produces cumulative toxicity yet rarely durable remission. We hypothesised that refractory AIC reflects failed immune reconstitution driven by pharmacologic immunosuppression impairing regulatory T-cell (Treg) development, and that withdrawing immunosuppression might permit endogenous tolerance recovery.

**Methods:**

We reviewed 20 consecutive children with refractory AIC after allo-HSCT (2019–2025). All received corticosteroids (± IVIG) and rituximab. Non-responders underwent withdrawal of all immunosuppressants, including calcineurin inhibitors (Step 1, n=5). Those who failed Step 1 or presented with life-threatening deterioration were allocated to parallel second-line strategies based on cryopreserved donor cell availability: PD-1 inhibitors ± interferon-alpha2b (Step 2, n=8) or donor lymphocyte infusion (Step 3, n=7). The primary endpoint was sustained remission; the secondary endpoint was optimal outcome (sustained remission without relapse and without Grade II–IV graft-versus-host disease [GVHD]).

**Results:**

All 20 children achieved sustained remission (100%; 95% CI 83.2–100%) at a median follow-up of 22.2 months. Optimal outcomes occurred in 80% of Step 1 patients versus 62.5% in Step 2 and 28.6% in Step 3 (Cochran-Armitage trend test, P = 0.070). GVHD incidence increased with treatment intensity: 20% after Step 1, 50% after Step 2, and 100% after Step 3 (P<0.001); all events were acute and self-limited.

**Conclusion:**

All children achieved sustained remission with this stepwise protocol, and the most favourable outcomes occurred in the group that withdrew immunosuppressants alone. Because treatment was not randomised, this apparent advantage may reflect patient selection rather than true efficacy. These observations are exploratory and require prospective validation.

## Introduction

Autoimmune cytopenia (AIC) after allogeneic hematopoietic stem cell transplantation (allo-HSCT) poses a significant clinical challenge: it is uncommon enough that prospective trials are rarely feasible, yet severe enough that refractory cases carry substantial mortality (1–4). In pediatric cohorts, incidence ranges from 2.4% to 6%, with autoimmune hemolytic anemia (AIHA) as the dominant phenotype (2, 3, 5–7). Single-lineage disease (AIHA or immune thrombocytopenia [ITP]) and multi-lineage involvement (Evans syndrome) both occur (2, 3, 6). Risk factors—younger age, nonmalignant disease, unrelated donors, and T-cell-depleting conditioning—converge on a shared mechanism: dysregulated immune reconstitution (8–11). Despite this mechanistic insight, therapeutic advances have lagged behind.

The core therapeutic dilemma is that roughly half of patients respond to conventional immunosuppression (steroids, IVIG, rituximab), while the other half do not (3, 12–14). For refractory cases, escalating immunosuppression remains the default, yet durable remission is uncommon and cumulative toxicity—including infection, endocrine damage, and avascular necrosis—is substantial (15–18). This creates a paradox: AIC may reflect failed immune reconstitution rather than conventional autoimmunity, suggesting that restoring immune balance by withdrawing—rather than deepening—immunosuppression could be a more rational strategy (9, 19), yet a structured framework for this approach has not been formally assessed.

From a mechanistic standpoint, calcineurin inhibitors (CNIs)—the backbone of graft-versus-host disease (GVHD) prophylaxis—block the calcium–calcineurin–NFAT axis required for both thymic negative selection and the induction of Foxp3^+^ regulatory T cells (Tregs) (20–22). Prolonged CNI exposure after allo-HSCT impairs *de novo* Treg generation in the thymus, suppresses naïve T-cell egress, and limits peripheral Treg expansion, thereby creating a permissive environment for autoreactive clone expansion. Programmed death-1 (PD-1) expression rises during T-cell exhaustion after lymphodepleting conditioning; while PD-1 signalling limits effector damage, persistent ligation can paralyse donor-derived immune surveillance and disrupt the Treg–effector balance (23–26). PD-1 blockade may therefore restore both effector function and Treg homeostasis in exhausted T-cell pools. Donor lymphocyte infusion (DLI), by contrast, supplements donor T-cell chimerism and can clear residual host autoreactive lymphocytes, particularly in the setting of mixed chimerism, while concurrently expanding the donor Treg compartment (27–29). These three modalities—CNI withdrawal, PD-1 inhibition, and DLI—thus represent distinct but complementary routes toward the same endpoint: restoration of donor-derived immune tolerance.

We adopted a stepwise protocol at our center. After initial treatment with corticosteroids (± IVIG) and rituximab, non-responders first underwent immunosuppressant withdrawal (Step 1). For those who remained refractory, treatment allocation depended on two practical considerations: cryopreserved donor stem cell availability and clinical disease severity. Patients with life-threatening manifestations—defined as haemoglobin <40 g/L with haemodynamic instability, platelets <10×10^9^/L with active bleeding, or rapidly progressive Evans syndrome—could be escalated to Step 2 or 3 within two to four weeks per clinical judgment. Among those requiring escalation, patients without cryopreserved donor cells received PD-1 inhibitors with or without interferon (IFN)-alpha2b guided by PD-1 expression levels (Step 2), whereas those with available cryopreserved cells received donor lymphocyte infusion (DLI) (Step 3). Steps 2 and 3 thus ran in parallel, reflecting a practical pairing of two immunomodulatory strategies rather than a strict sequential escalation ladder.

We report the outcomes of 20 consecutive children with refractory AIC after allo-HSCT managed with this institutional stepwise protocol in a retrospective case series. The primary objective was to describe durable remission rates; the secondary objective was to assess toxicity by treatment modality and to explore, in a subset of patients with available samples, whether immunophenotypic changes in regulatory T cells and PD-1 expression might inform mechanistic hypotheses. Because the series is retrospective, non-randomised, and small, any differences between steps should be viewed as exploratory, not proof that one strategy is superior to another. The practical diagnostic and therapeutic algorithm is summarised in **Figure 1**.

**Figure 1:**
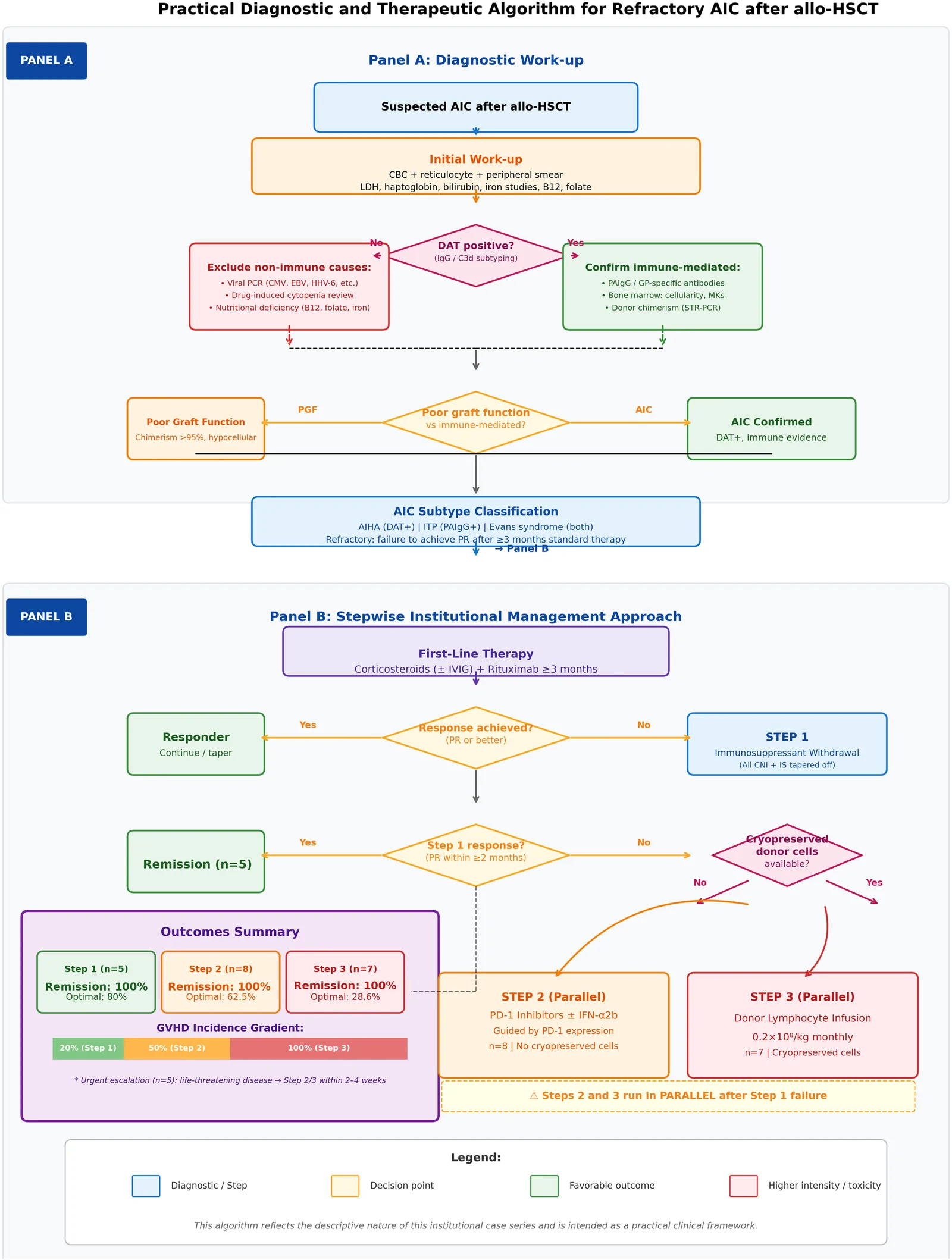
Practical diagnostic and therapeutic algorithm for refractory autoimmune cytopenia (AIC) after allogeneic hematopoietic stem cell transplantation (allo-HSCT). **(A)** Diagnostic work-up to distinguish immune-mediated cytopenia from poor graft function, viral infection, drug toxicity, and nutritional deficiency. Key investigations include direct antiglobulin test (DAT), platelet-associated immunoglobulin (PAIgG), bone marrow examination, donor chimerism analysis, and viral PCR panel. **(B)** Stepwise institutional management approach. All patients receive first-line corticosteroids (± IVIG) and rituximab. Non-responders proceed to Step 1 (immunosuppressant withdrawal). Patients refractory to Step 1, or those with life-threatening deterioration requiring concurrent Step 1 and escalation, are allocated to parallel second-line strategies based on cryopreserved donor stem cell availability: Step 2 (PD-1 inhibitors ± interferon-alpha2b guided by PD-1 expression) for those without available cells, or Step 3 (donor lymphocyte infusion) for those with available cells. The figure reflects the descriptive nature of this institutional case series and is intended as a practical clinical framework rather than an evidence-based treatment guideline.

## Methods

### Study design and ethics

We reviewed the records of 20 consecutive children treated for refractory AIC after allo-HSCT at Beijing JingDu Children’s Hospital between January 2019 and May 2025. This is a retrospective case series; there was no control group, and the choice of treatment step depended on clinical need rather than randomization.

All treatment decisions were made by the treating physicians in real time, independent of and before this retrospective analysis. The stepwise protocol remained unchanged throughout the study period. PD-1 inhibitors, interferon-alpha2b, and donor lymphocyte infusion were all available throughout, and the same multidisciplinary team applied the same criteria to every patient.

The institutional ethics committee approved retrospective review on February 28, 2026 (No. 2026-001-C) and waived informed consent under applicable regulations for minimal-risk research using fully de-identified data. Personal identifiers were removed before data extraction; only sequential study numbers link clinical records to immunophenotyping samples.

### Patient population and refractory criteria

Eligibility required documented allo-HSCT and subsequent refractory AIC. We excluded patients with positive direct antiglobulin test (DAT) but nonimmune haemolysis (e.g., microangiopathic haemolytic anemia), primary or secondary poor graft function, and those lacking DAT (assumed no clinically significant AIHA). The diagnostic work-up to distinguish immune-mediated cytopenia from poor graft function and other post-transplant causes included the following systematic evaluation: (i) complete blood count with reticulocyte count and peripheral blood smear review; (ii) direct antiglobulin test (DAT) with IgG and C3d subtyping for suspected AIHA; (iii) platelet-associated immunoglobulin (PAIgG) and glycoprotein-specific antibody testing for suspected ITP when available (30); (iv) bone marrow aspiration and biopsy to assess cellularity, megakaryocyte number, and erythroid hyperplasia; (v) donor chimerism analysis by short tandem repeat (STR) PCR to exclude poor graft function or graft rejection; (vi) viral PCR panel (CMV, EBV, HHV-6, adenovirus, parvovirus B19) to exclude viral-mediated cytopenia; (vii) iron studies, vitamin B12, and folate levels to exclude nutritional deficiency; (viii) lactate dehydrogenase (LDH), haptoglobin, and bilirubin for haemolysis work-up; and (ix) review of all medications for drug-induced cytopenia. Poor graft function was defined as persistent cytopenia beyond day +28 with complete or near-complete donor chimerism (>95%) and hypocellular or normocellular marrow without evidence of immune-mediated destruction. Patients with mixed chimerism and cytopenia were evaluated for graft rejection versus immune-mediated cytopenia based on marrow morphology, chimerism trends, and serologic evidence of autoimmunity. We diagnosed Evans syndrome when ITP and AIHA (DAT-positive) occurred simultaneously or sequentially.

We defined refractory AIC as failure to achieve partial response (PR) after ≥3 months of standard immunosuppressive therapy (steroids, ± IVIG and rituximab). Two pathways applied. In the standard pathway (n=15), patients completed ≥3 months of standard therapy without PR before entering Step 1. In the urgent escalation pathway (n=5), patients with life-threatening manifestations—haemoglobin <40 g/L with haemodynamic instability, platelets <10×10^9^/L with active bleeding, or rapidly progressive Evans syndrome—began Step 1 concurrently with Step 3 within two to four weeks per clinical judgment. All 20 patients entered the primary analysis; sensitivity analyses excluding urgent-escalation cases assessed robustness.

### Treatment strategy

All patients initially received corticosteroids (± IVIG) plus rituximab. Non-responders then entered Step 1: discontinuation of all immunosuppressants, including calcineurin inhibitors. Five patients (25%) responded to Step 1 alone. The remaining 15 underwent allocation based on two practical considerations: (i) cryopreserved donor stem cell availability and (ii) disease severity at the time of escalation decision. Step 2 and Step 3 were initiated after documented failure of Step 1, or concurrently with Step 1 for patients with life-threatening deterioration in whom waiting for Step 1 failure was deemed unsafe. In urgent cases, immunosuppressant withdrawal (Step 1) was begun simultaneously with Step 2 or 3 allocation.

Patients with available cryopreserved cells received donor lymphocyte infusion (DLI) (Step 3, n=7); those without available cells received PD-1 inhibitors with or without interferon-alpha2b guided by PD-1 expression levels (31) (Step 2, n=8). Steps 2 and 3 thus ran in parallel, reflecting a practical pairing of two immunomodulatory strategies rather than a strict sequential escalation ladder.

Step 2 (n=8): PD-1 inhibitors (1 mg/kg IV, one to two administrations per month), with or without interferon (IFN)-alpha2b (32) (100–300×10^4^ U/m^2^ SC twice weekly) guided by PD-1 expression levels (normal/low: IFN-alpha2b + PD-1 inhibitor; high: PD-1 inhibitor alone). For patients without cryopreserved donor stem cells.

Step 3 (n=7): Donor lymphocyte infusion (0.2×10^8^/kg monthly). For patients with available cryopreserved donor stem cells.

These durations refer to active drug administration; the time from escalation to sustained remission confirmation was longer due to required post-treatment follow-up (**Figure 2**).

**Figure 2:**
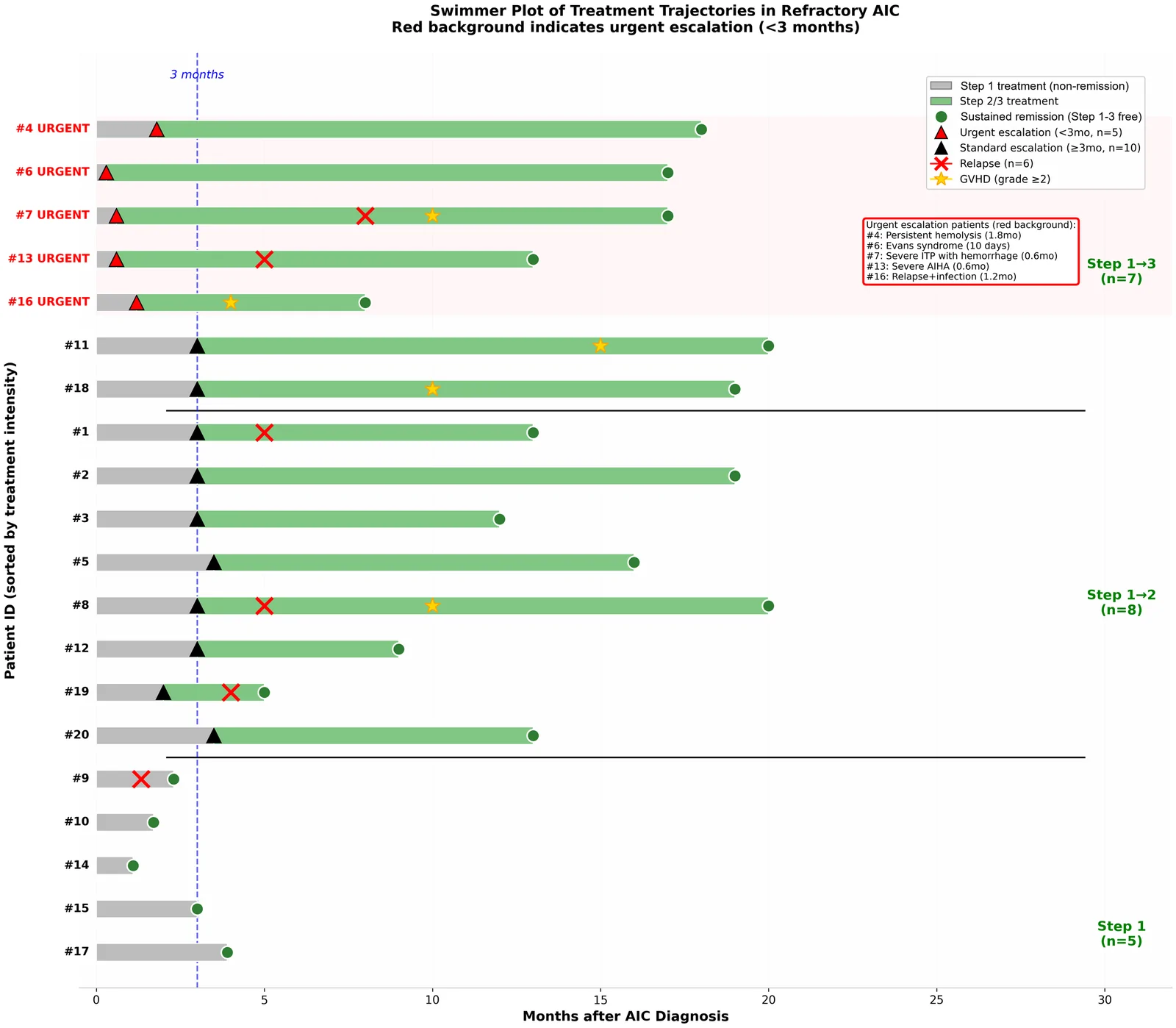
Swimmer plot of individual treatment trajectories. Each row represents one patient. Gray bars indicate Step 1 treatment (immunosuppressant withdrawal). Green bars indicate the period from escalation to sustained remission confirmation (including post-treatment follow-up), not active treatment duration alone. Patients requiring urgent escalation are highlighted. GVHD events (yellow stars), relapses (red crosses), and final outcomes are annotated.

### GVHD prophylaxis and immunosuppressive therapy at AIC onset

All 20 patients received calcineurin inhibitor (CNI)-based GVHD prophylaxis from transplant. Twelve (60%) received cyclosporin A (CSA) and eight (40%) received tacrolimus. CSA was started at 3 mg/kg/day intravenously, with trough levels targeted at 150–250 ng/mL for the first 100 days, then tapered to 100–150 ng/mL. Tacrolimus was started at 0.03 mg/kg/day intravenously, with trough levels targeted at 10–15 ng/mL for the first 100 days, then tapered to 5–10 ng/mL. All patients also received short-course methotrexate (15 mg/m^2^ on day +1, 10 mg/m^2^ on days +3, +6, and +11). Six patients (30%) with haploidentical donors or cord blood transplants also received mycophenolate mofetil (MMF, 15 mg/kg twice daily, maximum 2 g/day). No patient received sirolimus or post-transplant cyclophosphamide.

When AIC developed, all 20 patients were still on maintenance CNI (CSA in 11, tacrolimus in 9). The median time from transplant to AIC onset was 9 months (range 4–67). At that point, 18 patients (90%) were also taking corticosteroids (prednisone 0.5–1 mg/kg/day or equivalent), four (20%) were on MMF, and one (5%) was on low-dose azathioprine. Step 1 involved tapering and stopping all of these agents; CNI withdrawal took a median of 2 weeks (range 1–4 weeks), guided by clinical response and careful GVHD monitoring.

### Data collection

Demographics, underlying disease, AIC subtype and chronology, donor type, conditioning regimen, cell doses, GVHD occurrence, treatment response, and duration were extracted from inpatient and outpatient records.

### Treatment response evaluation

We defined four response levels, incorporating transplant-specific requirements (sustained donor chimerism, no active GVHD):

Sustained remission: Complete normalization of blood counts with resolution of haemolytic markers (negative DAT, normal bilirubin, LDH, and reticulocytes); no immunosuppressive therapy for ≥6 months; no active GVHD with stable donor chimerism.

Complete response (CR): Normalization of blood counts (haemoglobin ≥110 g/L, platelets ≥100×10^9^/L, ANC >1.5×10^9^/L) with normal haemolytic markers; transfusion independence; low-dose maintenance therapy permitted.

Partial response (PR): Improved but not fully normalized blood counts (haemoglobin ≥80 g/L or ≥20 g/L above baseline, platelets ≥30×10^9^/L, ANC ≥0.5×10^9^/L); improved haemolytic markers; transfusion independence for ≥7 consecutive days.

Refractory: Failure to achieve partial response (PR) after ≥3 months of standard therapy.

Optimal outcome: Sustained remission without relapse and without Grade II–IV acute graft-versus-host disease. Grade I GVHD—limited to skin rash involving <25% body surface area, liver bilirubin 2–3 mg/dL, or mild gastrointestinal symptoms not requiring systemic corticosteroids—was not considered a treatment failure, because all Grade I events in this series were transient and self-limited, resolving without systemic therapy or within 2 weeks of brief low-dose corticosteroids.

### Statistical analysis

We expressed categorical variables as frequency (%) with exact 95% confidence intervals (Clopper-Pearson) and continuous variables as median (range). For three-group comparisons of categorical outcomes, we performed pairwise Fisher’s exact tests with Bonferroni correction (adjusted alpha = 0.0167 for three comparisons). The Cochran-Armitage trend test assessed the association between ordered treatment intensity and binary outcomes (optimal outcome, GVHD incidence). We report two-sided P values without continuity correction; with continuity correction, P values were slightly larger and are provided for sensitivity. We set two-sided p < 0.05 as significant. We also calculated the fragility index for the primary outcome.

Competing risk analysis. We used the Fine-Gray model to estimate the cumulative incidence of GVHD and sustained remission over time. Strictly speaking, these are not competing events in the conventional sense, because the occurrence of GVHD did not preclude subsequent sustained remission—all patients who developed GVHD were ultimately salvaged. Rather than treating them as mutually exclusive outcomes, we applied the Fine-Gray approach to describe how the probability of each event accumulated while accounting for the fact that, once a patient achieved sustained remission, they were no longer at risk for the other endpoint. Subdistribution hazard ratios (sHR) with 95% confidence intervals are reported for descriptive purposes only. We assessed baseline balance using standardised mean differences (SMD); values >0.2 indicated potential imbalance.

### Flow cytometry and regulatory T-cell assessment

Peripheral blood samples were collected at clinically indicated time points relative to immunomodulation therapy. Immunophenotyping was performed on a Beckman Coulter DxFLEX flow cytometer using CytExpert for DxFLEX software (Beckman Coulter, Brea, CA, USA). Samples were stained with fluorochrome-conjugated antibodies against CD3, CD4, CD8, CD19, CD16, CD56, CD25, and CD127 (Beckman Coulter). Regulatory T cells were defined as CD4^+^CD25^+^CD127_r_^o^⊔^−^ cells and expressed as a percentage of total CD4^+^ T cells. Programmed death-1 (PD-1) expression was assessed on CD4^+^ and CD8^+^ T cells and reported as percentage of positive cells. Reference ranges for healthy children were obtained from published literature.

Longitudinal Treg and PD-1 data were available for 7 of 20 patients (35%) for exploratory analysis. These data were not used to guide treatment decisions but were analysed retrospectively to generate mechanistic hypotheses. Given the small sample size and exploratory nature, no formal statistical comparisons were performed for immunophenotyping data.

## Results

### Patient characteristics

We included 20 children (13 male, 7 female) with a median age of 4 years (range 1.25–17). Underlying diseases varied: aplastic anemia (n=5, 25%), chronic granulomatous disease (n=4, 20%), haemophagocytic lymphohistiocytosis (n=3, 15%), Wiskott–Aldrich syndrome (n=2, 10%), mucopolysaccharidosis (n=2, 10%), and one each with congenital neutropenia, B-cell acute lymphoblastic leukemia, pyruvate kinase deficiency anemia, and thalassaemia. Donor sources were haploidentical (n=10, 50%), cord blood (n=7, 35%), matched sibling (n=2, 10%), and matched unrelated (n=1, 5%). AIC developed at a median of 9 months post-transplant (range 4–67). Patients had a median of 2 AIC episodes (range 1–5). Five patients (25%) entered the urgent-escalation pathway due to life-threatening disease. **Table 1** shows baseline characteristics by treatment group; individual details are in **Supplementary Table 3B**.

**Table 1:** Baseline characteristics stratified by treatment group.

Characteristic	Step 1 (n=5)	Step 2 (n=8)	Step 3 (n=7)	SMD
Age, median (range), y	3 (1.25-16)	4.5 (1.4-10)	4 (1.4-17)	0.12
Male sex, n (%)	4 (80%)	5 (62.5%)	4 (57.1%)	0.28
Primary disease, n (%)				
Aplastic anemia	1 (20%)	2 (25%)	2 (28.6%)	0.13
Chronic granulomatous disease	0 (0%)	2 (25%)	2 (28.6%)	0.50
Hemophagocytic lymphohistiocytosis	2 (40%)	1 (12.5%)	0 (0%)	0.63
Wiskott-Aldrich syndrome	1 (20%)	0 (0%)	1 (14.3%)	0.28
Mucopolysaccharidosis	1 (20%)	1 (12.5%)	0 (0%)	0.35
Other^a	0 (0%)	2 (25%)	2 (28.6%)	0.54
AIC type, n (%)				
AIHA	2 (40%)	5 (62.5%)	3 (42.9%)	0.31
ITP	2 (40%)	2 (25%)	1 (14.3%)	0.28
Evans syndrome	1 (20%)	1 (12.5%)	3 (42.9%)	0.54
Time to AIC, median (range), mo	12 (4-67)	8.5 (4-15)	9 (4-14)	0.18
Recurrent episodes, median (range)	1 (1-3)	3 (2-5)	2 (1-5)	0.71
Donor source, n (%)				
Haploidentical	2 (40%)	4 (50%)	4 (57.1%)	0.13
Cord blood (UCBT)	3 (60%)	3 (37.5%)	1 (14.3%)	0.67
Matched sibling (MSD)	0 (0%)	0 (0%)	2 (28.6%)	0.98
Matched unrelated (MUD)	0 (0%)	1 (12.5%)	0 (0%)	0.35
Conditioning with ATG, n (%)	5 (100%)	8 (100%)	7 (100%)	0.00
Prior treatment lines, median (range)	3 (2-4)	3 (2-4)	3 (2-5)	0.05

Data are n (%) or median (range). SMD, standardised mean difference; AIC, autoimmune cytopenia; AIHA, autoimmune hemolytic anemia; ITP, immune thrombocytopenia; UCBT, umbilical cord blood transplantation; MSD, matched sibling donor; MUD, matched unrelated donor; ATG, antithymocyte globulin. ^a^Other, congenital neutropenia, B-ALL, pyruvate kinase deficiency anemia, thalassaemia.

### Treatment administration

All 20 children received antithymocyte globulin-containing conditioning. Five (25%) underwent urgent escalation to Step 3 within three months due to life-threatening severity; all five concurrently began immunosuppressant withdrawal (Step 1). Two had Evans syndrome with rapid progression, two had severe AIHA with haemoglobin <40 g/L, and one had severe ITP with active bleeding. Step 1 (n=5) involved immunosuppressant withdrawal alone for a median of 2 months (range 0.5–4). Step 2 (n=8) comprised PD-1 inhibitor monotherapy (n=2) or interferon-alpha2b plus PD-1 inhibitor (n=6) for a median of 3 months (range 1–8). Step 3 (n=7) used DLI alone (n=6) or DLI plus PD-1 inhibitor (n=1) for a median of 3 months (range 2–6). Individual treatment details are in **Supplementary Table 3B**.

### Treatment outcomes

Treatment outcomes and safety by group are summarised in **Table 2**. Fourteen achieved sustained remission directly; six relapsed (median time to relapse 4.5 months; range 1.3–8.0) but were subsequently salvaged.

**Table 2:** Treatment outcomes and safety by group.

Outcome	Step 1 (n=5)	Step 2 (n=8)	Step 3 (n=7)	P-value
**Follow-up, median (range), mo**	**22.2 (8.6–78.0)**			
Sustained remission, n (%)	5 (100%)	8 (100%)	7 (100%)	
**Direct sustained remission**	**4 (80%)**	**5 (62.5%)**	**5 (71.4%)**	
**Relapse then salvaged**	**1 (20%)**	**3 (37.5%)**	**2 (28.6%)**	
**Sustained remission rate, % (95% CI)**	**100 (83.2–100)**	**100 (63.1–100)**	**100 (59.0–100)**	
**Optimal outcome^a^, n (%)**	**4 (80%)**	**5 (62.5%)**	**2 (28.6%)**	**0.070^b^**
Relapse, n (%)	1 (20%)	3 (37.5%)	2 (28.6%)	
Time to relapse, median (range), mo	4.5 (1.3–8.0)			
GVHD, n (%)	1 (20%)	4 (50%)	7 (100%)	<0.001
Grade I	1 (20%)	3 (37.5%)	3 (42.9%)	
Grade II	0 (0%)	1 (12.5%)	4 (57.1%)	
Grade III–IV	0 (0%)	0 (0%)	0 (0%)	
GVHD organ involvement, n				
**Skin**	**0**	**4**	**6**	
**Liver**	**1**	**1**	**3**	
GI tract	0	0	0	
New infections, n (%)	2 (40%)	3 (37.5%)	5 (71.4%)	0.224
Upper respiratory tract infection	1	1	5	
CMV reactivation	0	2	0	
**COVID-19**	**1**	**0**	**0**	

Optimal outcome = sustained remission without relapse and without Grade II–IV GVHD. Grade I GVHD—limited to skin rash involving <25% body surface area, liver bilirubin 2–3 mg/dL, or mild gastrointestinal symptoms not requiring systemic corticosteroids—was not considered a treatment failure. GVHD = graft-versus-host disease. P for trend from Cochran–Armitage test (two-sided, without continuity correction; with continuity correction, P = 0.128) for categorical variables. Detailed GVHD characteristics (grade, organ involvement, treatment) and infection types are described in the Results. Bold values indicate the primary endpoint (sustained remission), secondary endpoint (optimal outcome), and key outcome/safety measures (relapse and GVHD).

### GVHD characteristics

All GVHD events were acute and occurred during the immunomodulation period. In Step 1, one patient developed Grade I liver aGVHD that resolved without treatment. In Step 2, three patients had self-limited Grade I skin aGVHD and one developed Grade II skin and/or liver aGVHD requiring brief low-dose corticosteroids (resolved within two weeks). In Step 3, three patients had self-limited Grade I skin aGVHD and four had Grade II aGVHD involving the skin and/or liver; three required corticosteroids and one resolved without treatment. No Grade III–IV GVHD occurred and no patient died from GVHD. All events resolved fully without progression to chronic GVHD or need for second-line therapy. Every patient who developed GVHD—including those with Grade II disease—achieved sustained remission, consistent with the interpretation that mild-to-moderate GVHD in this setting may reflect graft-versus-autoimmunity.

### Infectious complications

Patient 9 experienced transient relapse at 1.5 months following COVID-19 but was salvaged without interrupting the withdrawal protocol; both infections were managed conservatively without interrupting treatment.

### Competing risk analysis

The Fine-Gray model (**Figure 3**) showed two patterns. Panel A displays cumulative incidence of sustained remission from AIC diagnosis: all patients achieved sustained remission, with Step 1 showing the shortest median time (2.3 months) and Step 3 the longest (16.2 months). The apparent delay in Step 2 and Step 3 reflects the Step 1 observation period before escalation. Compared with Step 1, the subdistribution hazard ratio for sustained remission was 0.4 (95% CI 0.1–1.5) in Step 2 and 0.3 (95% CI 0.1–1.3) in Step 3, reflecting slower remission rates with increasing treatment intensity. Panel B shows cumulative GVHD incidence from AIC diagnosis: probability of GVHD rose steadily with treatment intensity, reaching 20% in Step 1 (at 1.5 months), 50% in Step 2 (by 13 months), and 100% in Step 3 (by 17 months). Each curve plateaued after the initial rise, consistent with the clinical observation that all GVHD episodes were acute and self-limited with no chronic progression. The Cochran-Armitage trend test confirmed a significant association between treatment intensity and GVHD incidence (Z = 2.87, p for trend <0.001).

**Figure 3:**
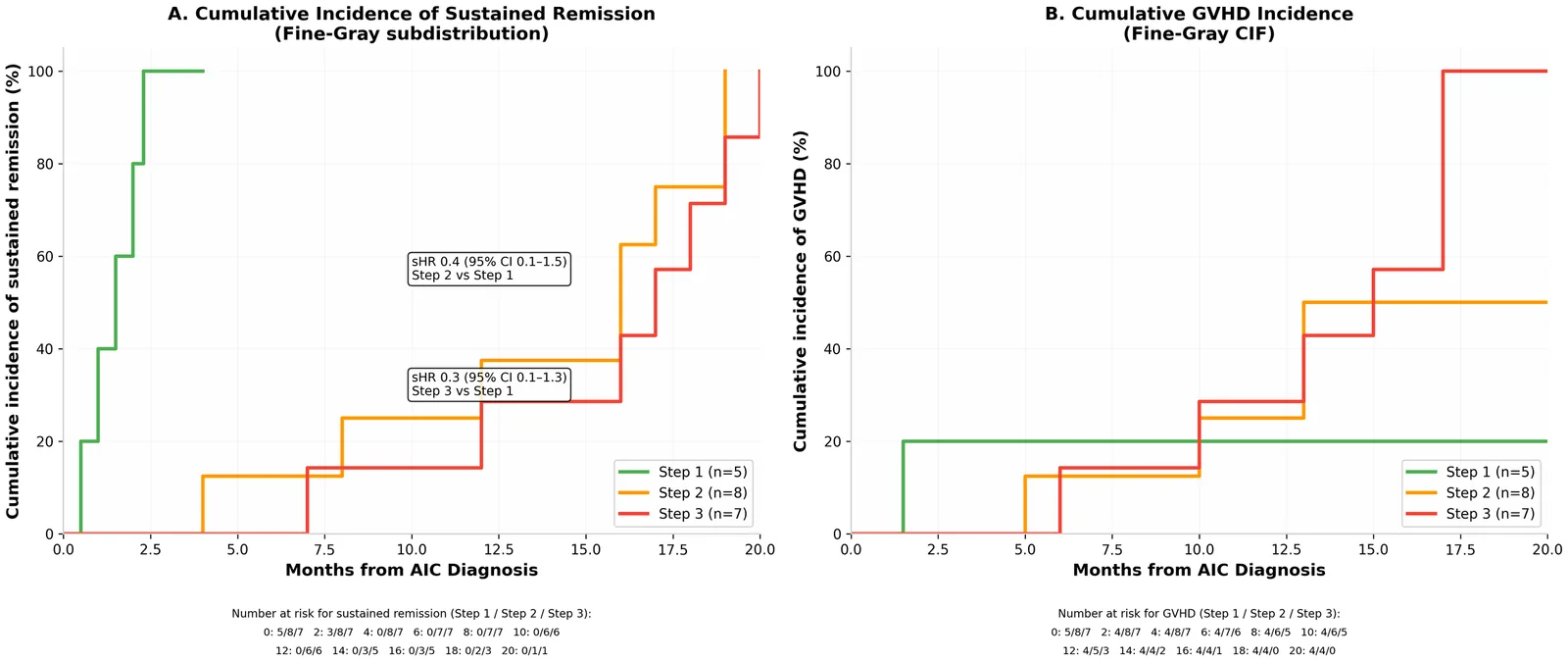
Competing risk analysis of immunomodulation therapy. **(A)** Cumulative incidence of sustained remission estimated by Fine-Gray subdistribution analysis; time 0 = AIC diagnosis for all patients. Step 2 and Step 3 curves include the Step 1 observation/withdrawal period. Subdistribution hazard ratios for Step 2 and Step 3 versus Step 1 are shown for descriptive comparison. **(B)** Cumulative incidence of GVHD estimated by Fine-Gray cumulative incidence function. Note that GVHD and sustained remission are not strictly competing events in this series because all patients who developed GVHD subsequently achieved sustained remission; the analysis therefore describes the temporal accumulation of each endpoint rather than comparing mutually exclusive outcomes. Number at risk tables appear below each curve. Step 1 (green), Step 2 (orange), Step 3 (red).

### Sensitivity analysis

After excluding the five urgent-escalation patients (all in Step 3, who had received concurrent Step 1), optimal outcome rates were 80% (Step 1; 4/5), 62.5% (Step 2; 5/8), and 0% (Step 3; 0/2). The between-group trend approached statistical significance in this sensitivity analysis (Cochran-Armitage trend test, Z = –1.78, P = 0.076; with continuity correction, P = 0.170). **Figure 2** displays individual treatment trajectories: all five urgent-escalation patients achieved sustained remission, and GVHD events clustered exclusively within the escalation groups. Clinical indications for urgent escalation were severe AIHA with persistent haemolytic anemia at 1.8 months (Patient 4), Evans syndrome at 10 days (Patient 6), severe ITP with haemorrhage at 0.6 months (Patient 7), severe AIHA at 0.6 months (Patient 13), and Evans syndrome with infection after steroid withdrawal at 1.2 months (Patient 16).

Additional sensitivity analyses supported the robustness of these findings. Stratification by AIC subtype (AIHA vs ITP vs Evans syndrome) yielded consistent optimal outcome gradients across all subtypes. Exclusion of the single patient with malignant disease did not materially alter the results. Donor source stratification (haploidentical vs cord blood vs matched sibling/unrelated) showed comparable patterns within each donor category. Reclassification of Grade I GVHD as non-significant reduced the absolute optimal outcome rates but preserved the between-group gradient. Collectively, these analyses suggest that the observed treatment-intensity gradient is not driven by a single patient characteristic or outlier.

### Exploratory immunophenotyping

In 7 patients (35%), longitudinal Treg and PD-1 data were available for exploratory analysis. These data should be interpreted with substantial caution given the small sample size, retrospective design, and the possibility that patients selected for immunophenotyping represented a distinct clinical subset. No formal statistical comparisons were performed.

Among 2 patients treated with Step 1 (immunosuppressant withdrawal) who had available immunophenotyping data, both showed Treg percentage increases after calcineurin inhibitor discontinuation. In Case 9 (ITP), Treg percentage rose from 2.37% to 5.51% during CSA tapering, concurrent with platelet recovery from 89 to 325 ×10^9^/L. In Case 1 (AIHA), Treg increased from 6.15% to 18.04% after CSA withdrawal, followed by clinical remission. These observations are descriptive only; with n = 2, no inference about the relationship between Treg recovery and clinical response can be drawn.

Among 4 patients treated with Step 2 (PD-1 inhibitors) with available immunophenotyping data, baseline PD-1 expression on T cells was elevated (CD4^+^PD-1^+^ 39.7–77.4%, CD8^+^PD-1^+^ 32.1–70.3%, exceeding reference ranges <20–42%). After PD-1 blockade, Treg percentages increased in 3 of 4 patients (from 2.53–3.44% to 5.23–6.65%), with clinical response observed in all 4. In Case 8, Treg rose from 2.53% to 6.65% but the patient developed Grade I skin GVHD. These observations are compatible with the hypothesis that PD-1 blockade may facilitate Treg recovery in T-cell exhaustion, but they cannot establish causality or identify PD-1 as a predictive biomarker.

In 1 patient treated with Step 3 (DLI, Case 13) with available immunophenotyping data, baseline Treg was 2.02% and rose to 22.75% after DLI (subsequently normalising to 5.48%), with concurrent PD-1 elevation (56.3%). The patient achieved sustained remission. This single observation is insufficient to draw any mechanistic conclusion about DLI-mediated immune modulation.

Collectively, these exploratory data are consistent with—but do not prove—the hypothesis that immunosuppressant withdrawal and PD-1 blockade may facilitate Treg recovery. Given the small sample size (n = 7, with only 2, 4, and 1 patients in Steps 1, 2, and 3 respectively), the absence of a systematic sampling strategy, and the lack of statistical comparisons, these observations serve solely to generate hypotheses for prospective validation.

## Discussion

### Principal finding

The most notable observation in this case series was that Step 1—withdrawal of immunosuppression—was associated with the most favourable outcomes. Four of five patients achieved sustained remission without relapse and without Grade II–IV GVHD. This observation, if confirmed in prospective studies, would be inconsistent with the usual practice of escalating immunosuppression when AIC proves refractory.

One plausible interpretation is that, in at least some patients, AIC may reflect failed immune reconstitution rather than classical autoimmunity, and that lifting pharmacologic suppression may permit endogenous tolerance mechanisms to recover. This interpretation must be tempered, however, by the strong possibility of confounding by indication: physicians likely assigned patients with milder disease to Step 1, so the superior outcomes may partly reflect patient selection rather than a pure biological effect. Baseline SMD reporting and sensitivity analyses address this concern only partially; confounding by indication remains the principal limitation of this observational study.

GVHD risk and relapse prevention did not appear to move together. GVHD incidence rose with treatment intensity (20% to 50% to 100%), but relapse rates did not show a consistent inverse relationship with treatment intensity (20%, 37.5%, 28.6%). This dissociation suggests potentially different mechanisms: GVHD likely reflects donor alloreactivity triggered by immune-activating interventions, whereas relapse may stem from transient immune dysregulation during incomplete reconstitution rather than from insufficient immunosuppressive intensity. In this cohort, adding PD-1 inhibitors or DLI increased toxicity without clear proportional gain in disease control.

### Mechanistic considerations

Post-transplant AIC is best understood as a disorder of immune reconstitution rather than conventional autoimmunity. Lymphocyte-depleting conditioning—whether with alemtuzumab or antithymocyte globulin—can disrupt T-cell/B-cell homeostasis, particularly when regulatory T-cell (Treg) preservation is inadequate, thereby triggering autoimmune manifestations (19, 33, 34). Three non-mutually exclusive mechanisms could explain why immunosuppressant withdrawal was associated with favourable outcomes in our cohort. First, calcineurin inhibitors, though necessary for GVHD prophylaxis, can impair thymic output and peripheral tolerance (20–22); their removal may permit regulatory T-cell recovery and restoration of self-tolerance. Second, withdrawal of pharmacologic suppression may allow activation-induced cell death to clear autoreactive clones that have expanded in the setting of impaired immune reconstitution. Third, restored thymic egress after calcineurin inhibitor cessation could replenish the Treg pool with naïve, polyclonal cells capable of re-establishing peripheral tolerance.

For patients who did not respond to withdrawal alone, Step 2 (PD-1 blockade) and Step 3 (DLI) represent two distinct immunomodulatory approaches. PD-1 blockade addresses T-cell exhaustion and can augment donor-derived immune activity (23–25), but early post-transplant PD-1 inhibition carries documented GVHD risk (24, 35). DLI can clear residual host autoreactive lymphocytes and reinforce donor chimerism, particularly in the setting of mixed chimerism (27, 28), though GVHD probability rises accordingly. In 7 patients (35%) with available longitudinal immunophenotyping data, exploratory observations were compatible with—but cannot establish—the tolerance-restoration hypothesis: Treg percentages appeared to rise after calcineurin inhibitor withdrawal in 2 of 2 patients with available Step 1 data, and after PD-1 blockade in 3 of 4 patients with available Step 2 data. Baseline PD-1 expression was elevated in these 4 patients, consistent with T-cell exhaustion. However, with only 2 patients contributing Step 1 immunophenotyping data and 4 contributing Step 2 data, these observations are purely hypothesis-generating. No causal inference can be drawn, and PD-1 expression cannot be considered a validated biomarker on the basis of these exploratory findings. Prospective studies with systematic Treg monitoring and predefined mechanistic endpoints are required to test these hypotheses.

### Comparison with existing literature

Our findings fit with what others have reported in children. Koo et al. found that only 25% of paediatric AIC patients respond to first-line therapy (36), which matches our experience that most children in this series needed stepwise salvage. Galvin et al. reported an 18% cumulative incidence of immune-mediated cytopenias after allo-HSCT for nonmalignant disorders, and 58% of their patients required prolonged immunosuppression (37). This background helps explain why we looked at immunosuppressant withdrawal rather than simply adding more drugs. Spadea et al. showed that rituximab can work but often causes long-term hypogammaglobulinemia requiring immunoglobulin replacement (38); less immunosuppressive options are therefore worth exploring. Prabahran et al. reviewed the difficulty of telling immune-mediated cytopenia apart from poor graft function and other non-relapse cytopenias (39), which is why we detailed our diagnostic algorithm in Methods. Baur et al. also pointed out that intensive immunosuppression increases infection and relapse risk, and that plasma cell-directed therapies, immunomodulation, and receptor-stimulating agents are emerging options (40). Conventional escalation strategies for refractory AIC have produced mixed results: plasma exchange and splenectomy have shown limited benefit (15, 16), and monoclonal antibodies or novel agents tested in small series have generally produced short-lived responses (17, 18). Our stepwise protocol-–starting with immunosuppressant withdrawal and adding PD-1 blockade when needed-–sits within this broader move away from blanket suppression.

Our approach differs from abatacept—which sustains immune suppression by blocking CD28 co-stimulation (41, 42)—and from adoptive Treg products such as Orca-T, which require complex manufacturing and specialised infrastructure (43, 44). In contrast, immunosuppressant withdrawal requires no additional pharmacologic agents or cellular products, and could be adopted in diverse clinical settings if validated prospectively.

More broadly, our observations align with emerging evidence that minimal intervention can achieve durable remission in selected post-transplant autoimmune complications. Recent reports of daratumumab for refractory post-HSCT immune-mediated cytopenias have shown promising responses (45–48), but accessibility and cost limit widespread adoption. A prospective single-centre study reported transfusion independence in 69% of patients with pure red cell aplasia at 6 months after daratumumab (46), and a multicentre cohort demonstrated 80% transfusion independence at 12 months (45). These data establish daratumumab as an effective salvage option, yet the median 88-day delay before initiating therapy underscores the need for earlier, less resource-intensive interventions.

The key challenge across all these approaches—including ours—is patient selection: identifying which patients will respond to minimal intervention and which require more aggressive modulation. Preliminary data suggest that PD-1 expression dynamics after allo-HSCT may serve as one such selection marker. In a study of 105 allo-HSCT recipients, PD-1 expression on CD4^+^ T cells was significantly elevated early after transplantation and remained persistently elevated, particularly in patients who received T-cell-depleting conditioning (49). These findings provide a mechanistic framework for our observation that patients with elevated baseline PD-1 expression responded to PD-1 blockade, and support the hypothesis that T-cell exhaustion—rather than conventional autoimmunity—underlies refractory AIC in a subset of post-transplant patients.

In this series every patient achieved sustained remission, whereas conventional escalation strategies have produced response rates of roughly 60–80% (3, 12, 13, 36–40). We do not claim our approach is superior. The comparison is crude: patient populations, treatment settings, and our retrospective non-randomised design differ too much to support any inference of superiority.

### Limitations

This was a retrospective, single-centre case series of 20 consecutive patients (fragility index = 1), so statistical power and generalisability are inherently limited. The study was designed to describe an institutional management approach and generate hypotheses, not to evaluate treatment efficacy or compare therapeutic superiority. All between-group comparisons should be interpreted as exploratory and hypothesis-generating, reflecting the non-randomised treatment allocation and the substantial risk of confounding by indication.

Treatment allocation followed clinical necessity, not randomisation, introducing confounding by indication that sensitivity analyses and SMD reporting can only partially offset. Although the stepwise protocol and treatment modalities remained consistent throughout the study period, individual variations in supportive care and timing of interventions may still exist as an inherent limitation of the retrospective design.

Immunophenotyping data were available for only 7 of 20 patients (35%); we cannot exclude the possibility that these 7 patients differed systematically from the 13 without such data. Patients selected for flow cytometric analysis may have represented a distinct clinical subset—for example, those with more complex disease courses or prolonged hospitalisations where additional testing was feasible.

We did not systematically capture immunological biomarkers; mechanistic inferences therefore rest on a combination of published literature and exploratory observations from a small patient subset. Infection surveillance was not standardised, so mild infections may have been missed, potentially underestimating the infectious burden of immunosuppressant withdrawal. The median 22.2-month follow-up is adequate for the primary endpoint but may miss very late relapses. Single-centre practice patterns in GVHD prophylaxis, donor selection, and supportive care may not apply elsewhere. All treatment decisions were made independent of and before this retrospective analysis; the ethics committee waived informed consent under applicable regulations for minimal-risk research using fully de-identified data.

### Future directions

Prospective multi-centre trials with systematic Treg monitoring and randomised assignment to early immunosuppressant withdrawal versus conventional escalation would clarify causality and identify the population most likely to benefit from minimal intervention. Such trials should incorporate predefined selection criteria—potentially including baseline PD-1 expression, Treg counts, and disease severity scores—to enable risk-stratified treatment allocation. Development of validated biomarker panels could eventually support bedside decision-making, allowing physicians to distinguish patients who need only immunosuppressant withdrawal from those who require immune-activating interventions.

## Conclusion

All 20 children in this small, retrospective, single-centre series achieved sustained remission with a stepwise protocol that began by withdrawing immunosuppressants. The best outcomes occurred in the group that received the least intensive treatment, but this probably reflects patient selection rather than true efficacy. We cannot claim that minimal intervention is superior to conventional escalation, because treatment was not randomised and the sample is far too small. Even so, our experience questions the reflex of adding more immunosuppression when first-line treatment fails. Whether a less-is-more approach can be validated will require prospective, multi-centre trials with clear entry criteria and mechanistic endpoints.

## Data Availability

The original contributions presented in the study are included in the article/**Supplementary Material**. Further inquiries can be directed to the corresponding authors.

## Supplementary material

Supplementary Table 3A Stratified baseline characteristics with standardised mean differences and individual patient-level data. SMD, standardised mean difference; SMD > 0.2 indicates potential between-group imbalance. AIHA, autoimmune hemolytic anemia; ITP, immune thrombocytopenia; UCBT, umbilical cord blood transplantation; MSD, matched sibling donor; MUD, matched unrelated donor. Key observations from SMD analysis: The most pronounced imbalances (SMD > 0.5) were observed for: (1) number of recurrent episodes (SMD = 0.71, Step 1 had fewer episodes than Step 2), (2) UCBT donor type (SMD = 0.67, Step 1 and 2 had more UCBT than Step 3), (3) MSD/MUD donor type (SMD = 0.83, exclusively in Step 3), and (4) hemophagocytic/metabolic disease category (SMD = 1.73, concentrated in Step 1). These imbalances support the possibility that patients assigned to Step 1 may have represented a less aggressive disease phenotype, reinforcing the confounding-by-indication concern discussed in the main text. The number of prior treatment lines was well-balanced (SMD = 0.05), suggesting comparable refractoriness at treatment initiation.

Supplementary Table 3B Individual patient-level treatment and outcome summary.
